# Supplementary material for: Lipid-mediated hydrophobic gating in the BK potassium channel
Source: Nat Commun. 2025 Aug 9;16:7354. doi: 10.1038/s41467-025-61638-9 (PMC12335557; doi:10.1038/s41467-025-61638-9)
Supplement: Supplementary file 1 — Supplementary Information [file 41467_2025_61638_MOESM1_ESM.pdf]

SUPPLEMENTARY MATERIALS
for

Lipid-mediated hydrophobic gating in the BK potassium channel

Lucia Coronel[1], Giovanni Di Muccio[2,4,*], Brad Rothberg[3], Alberto Giacomello[2], Enzo Carnevale[1,*]

*Corresponding authors: g.dimuccio@univpm.it, vincenzo.carnevale@temple.edu

[1]Institute for Computational Molecular Science and Institute for Genomics and Evolutionary Medicine and Department of Biology, Temple University, 1925 N. 12th St., Philadelphia, 19122, PA, USA.
[2]Department of Mechanical and Aerospace Engineering, Sapienza University of Rome, Via Eudossiana 18, Rome, 00184, Italy.
[3]Department of Medical Genetics and Molecular Biochemistry, Temple University, 3500 N. Broad St., Philadelphia, 19140, PA, USA.
[4]NY-Marche Structural Biology Center, Department of Life and Environmental Sciences, Marche Polytechnic University, Via Brecce Bianche, Ancona, 60131, Italy.

Contents:

Supplementary Figure S1: Deep Pore Volume definition. p. 2
Supplementary Figure S2: Equilibrium MD replicas. p. 3
Supplementary Figure S3: S6 helix orientation. p. 4
Supplementary Figure S4: S6 helix orientation data. p. 4-5
Supplementary Figure S5: Lipid-protein interaction frequencies. p. 6
Supplementary Figure S6: K392 Displacement. p. 7
Supplementary Figure S7: Number of water molecule during RMD sampling. p. 8
Supplementary Figure S8: Free-energy profile for WT Ca^{2+}-free (6V3G) state without intruded lipids. p. 9
Supplementary Figure S9: Comparison of TIP3P vs SPCE water model in RMD simulations. p. 10
Supplementary Figure S10: RMD of the full-length WT Ca^{2+}-int (8GHG) without intruded lipids. p. 11
Supplementary Figure S11: Top view of Fig. 4c snapshots. p. 12
Supplementary Figure S12: HOLE profiles of Fig. 4c snapshots. p. 13

Supplementary Table S1: Summary of helices S6 orientation data. p. 4

Supplementary Figures

FIGURE S1. - DPV

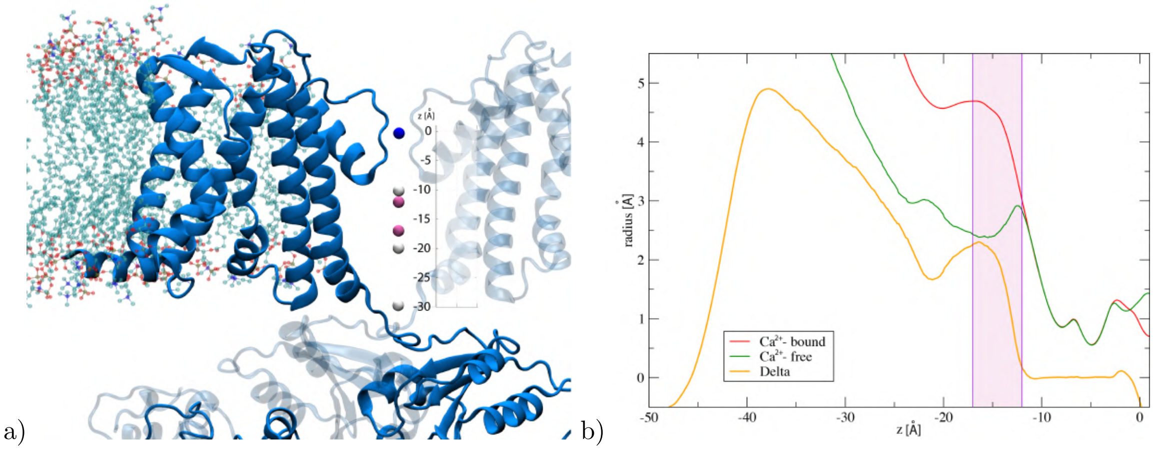

Fig. S1 a). Side view of the transmembrane portion in the WT Ca^{2+}-free state. The blue sphere indicates the selectivity filter center of mass (z=0 Å), the white spheres are located at z=-10,-20,-30 Å, and the pink ones at z=-12,-17 Å. For clarity, some subunits are not shown.

b) Average radius profiles for WT Ca^{2+}-free and WT Ca^{2+}-bound state. In orange is shown the difference Δ between WT Ca^{2+}-bound and WT Ca^{2+}-free radii. Notice that beneath the selectivity filter, Δ is equal to zero and starts increasing moving towards negative value of z, i.e. $z \sim -12$ Å, until it reaches a first maximum at $z \sim -17$ Å. These boundaries are chosen to define the Deep Pore Volume (DPV), here highlighted with a pink background.

FIGURE S2. - Long equilibrium MD replicas

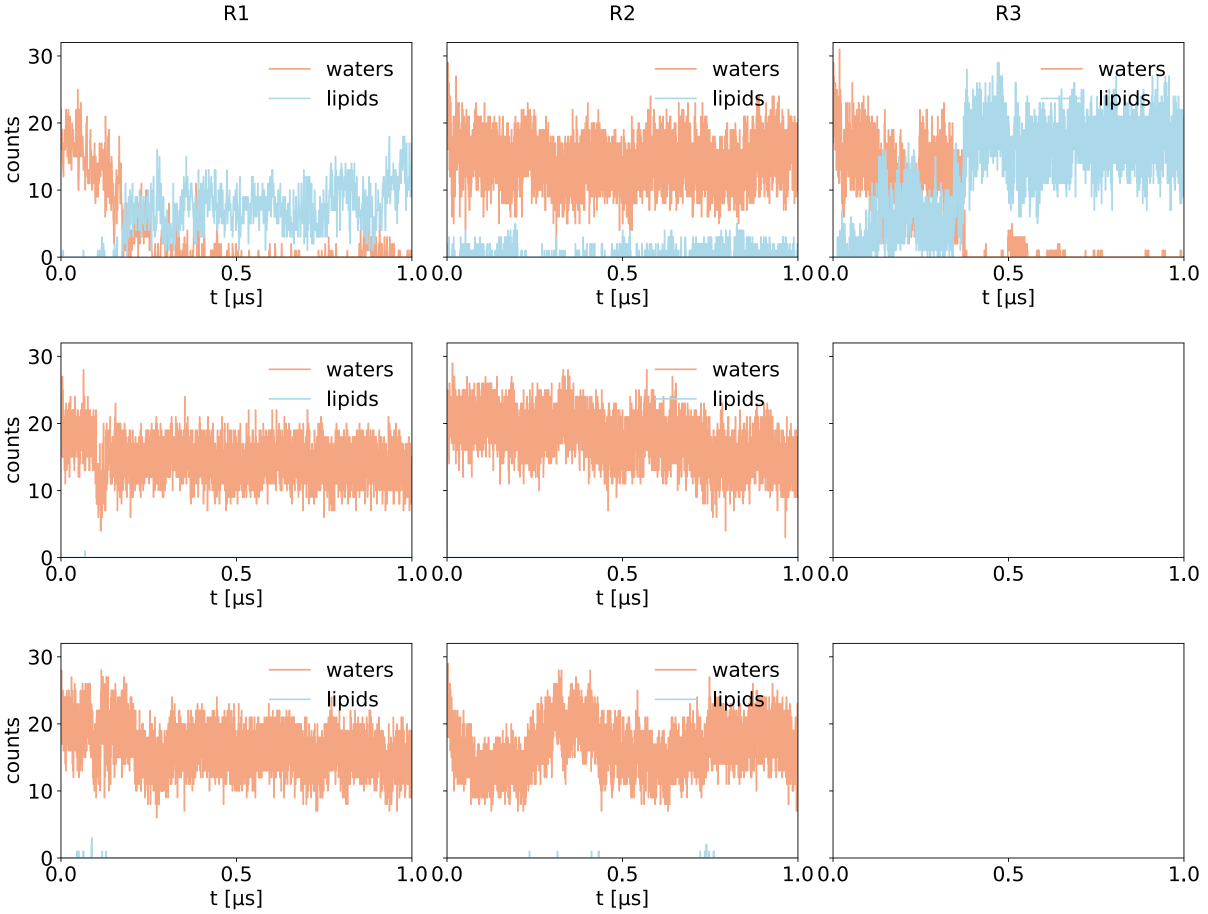

Fig. S2 Lipid carbon atoms (light-blue) and water molecules (orange) counts within the DPV for each replica: WT Ca^{2+}-free, WT Ca^{2+}-bound, and WT Ca^{2+}-int. Each replica has been generated independently using CHARMM-GUI web service [30, 32–35, 42, 55] and carried out following the protocol of described in Methods.

FIGURE S3. - Helices S6 orientation

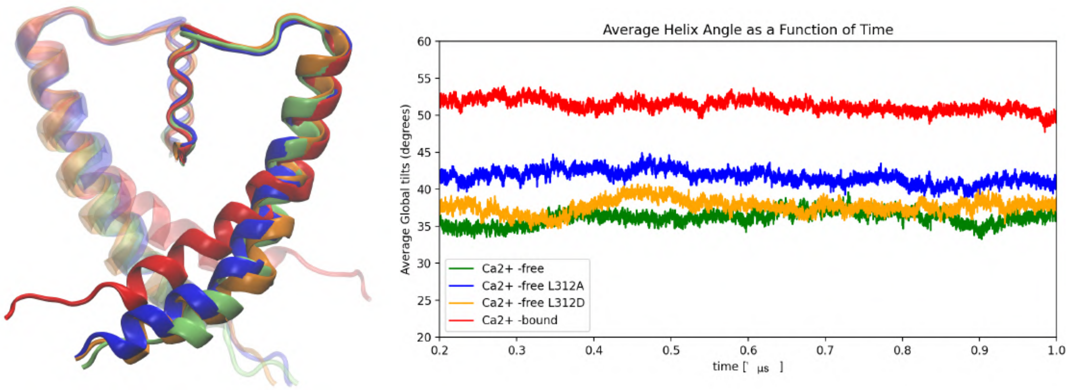

Fig. S3 Right. Side view of the S6 helices for wild type WT Ca^{2+}-free (green), L312A Ca^{2+}-free (blue), L312D Ca^{2+}-free (orange) and WT Ca^{2+}-bound (red). For clarity, only two subunits are shown. Left. Average angle between S6 and vertical axis as function of time.

We studied the S6 helices orientation with MDAnalysis HELANAL-routine [53, 54], and averaged the S6 angle with the vertical axis. The WT Ca^{2+}-free state results to be characterized by more elongated helices along the z-axis compared to WT Ca^{2+}-bound and the L312A mutant (Fig. S3).

FIGURE S4. - Helices S6 orientation data, WT Ca^{2+}-free and WT Ca^{2+}-bound

WT Ca^{2+}-free

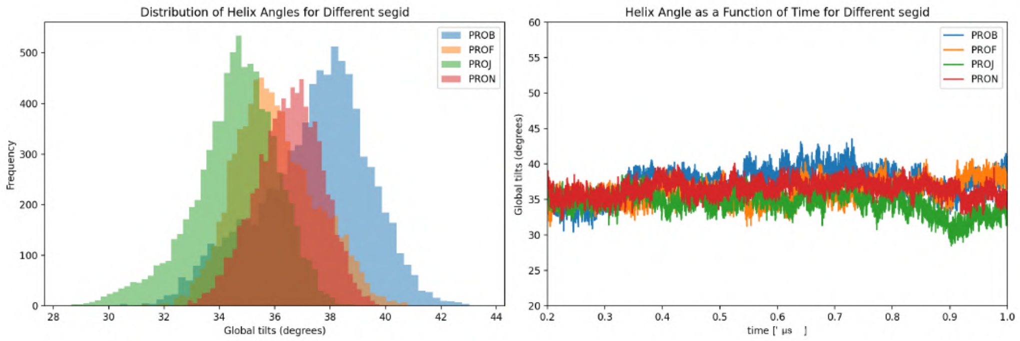

WT Ca^{2+}-bound

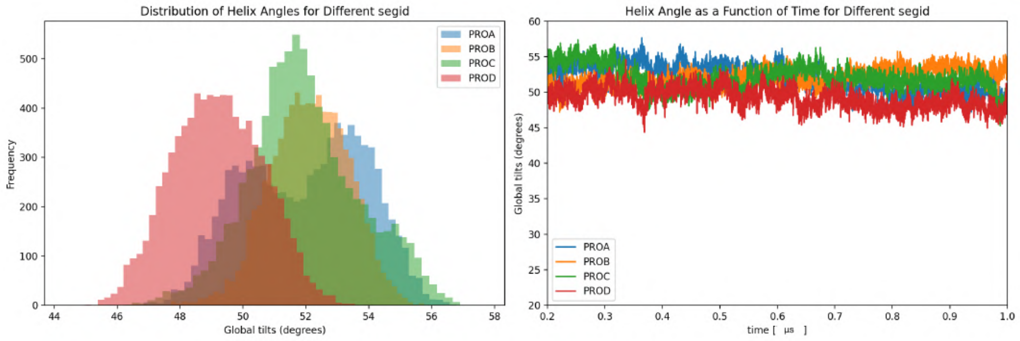

FIGURE S4.1 - Helices S6 orientation data, Mutants L312A,D

L312A Ca^{2+}-free

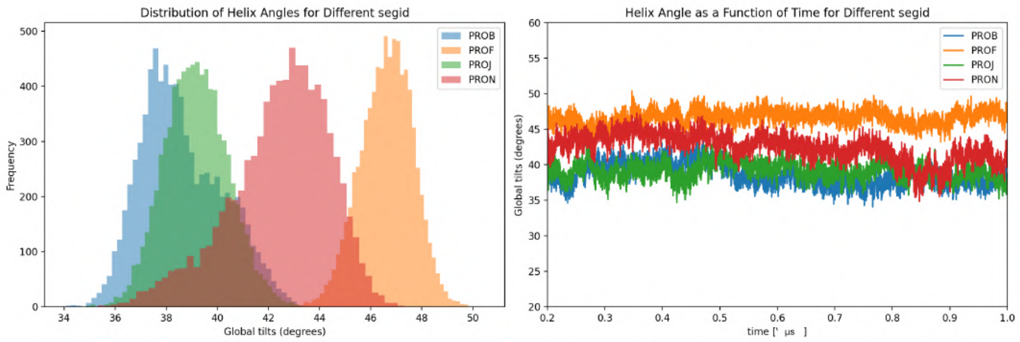

L312D Ca^{2+}-free

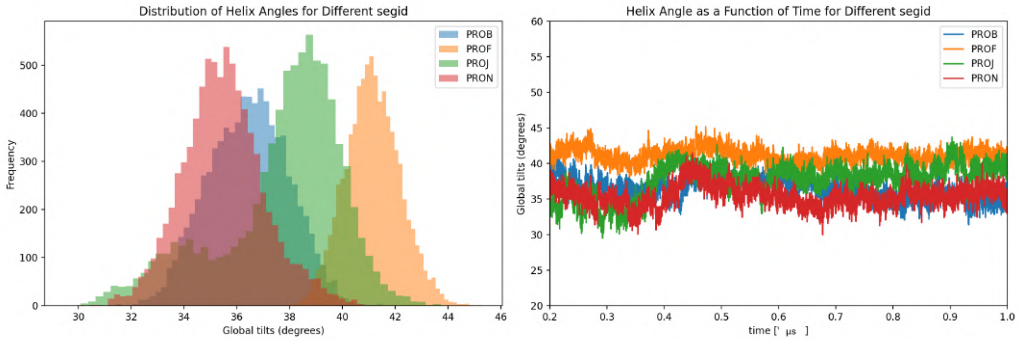

TABLE S1 - Helices S6 orientation data

**WT Ca^{2+}-free**		**WT Ca^{2+}-bound**		**L312A Ca^{2+}-free**		**L312D Ca^{2+}-free**	
**Mean**	**SD**	**Mean**	**SD**	**Mean**	**SD**	**Mean**	**SD**
36.866	2.160	52.268	2.138	38.292	1.682	36.460	1.489
35.876	1.486	52.203	1.273	46.015	1.847	40.948	1.197
34.639	1.567	52.063	1.750	38.973	1.449	37.023	2.596
36.947	1.781	49.337	1.593	42.228	1.968	35.081	1.728

Fig. S4 Summary statistics for S6-helix angles across different subunit and configurations: mean value and the sample standard deviation are listed for each case.

FIGURE S5. - Lipid-protein interactions

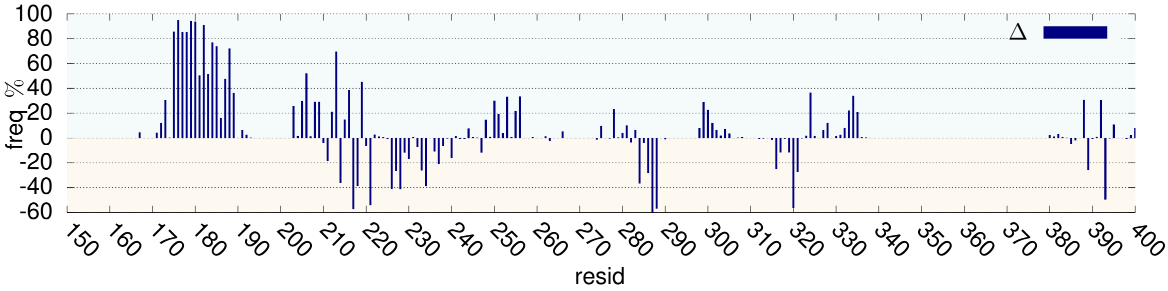

Fig. S5 The difference in interaction frequencies between the ^{2}+-bound and Ca^{2}+-free states is expressed as a percentage of the time each residue spends in contact (within 5Å) with lipids. For reference, S6 helices span from approximately residue 300 to 330, while S5 spans from 235 to 270. The blue background highlights where there is an excess of interactions for the WT Ca^{2+}-bound state, vice versa the yellow background shows interactions that are more frequent in the WT Ca^{2+}-free state compared to WT Ca^{2+}-bound state.

FIGURE S6. - K392 Displacement

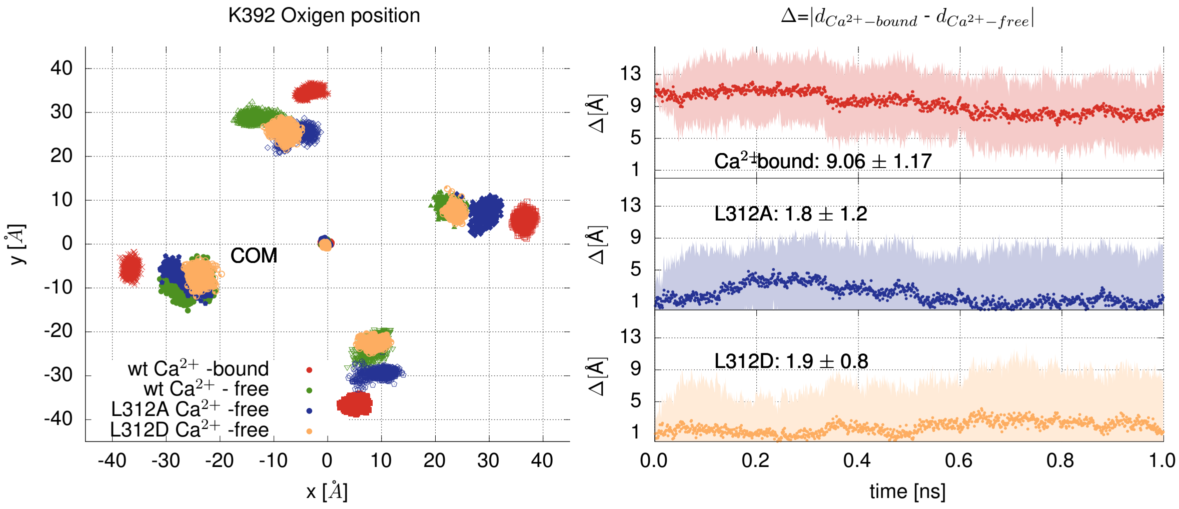

Fig. S6 Left. K392-Oxygen atom xy coordinates for both WT Ca^{2+}-free, WT Ca^{2+}-bound state, and mutants. Right. Displacement of K392-Oxygen atom compared with the WT Ca^{2+}-free.

FIGURE S7. - RMD trajectories

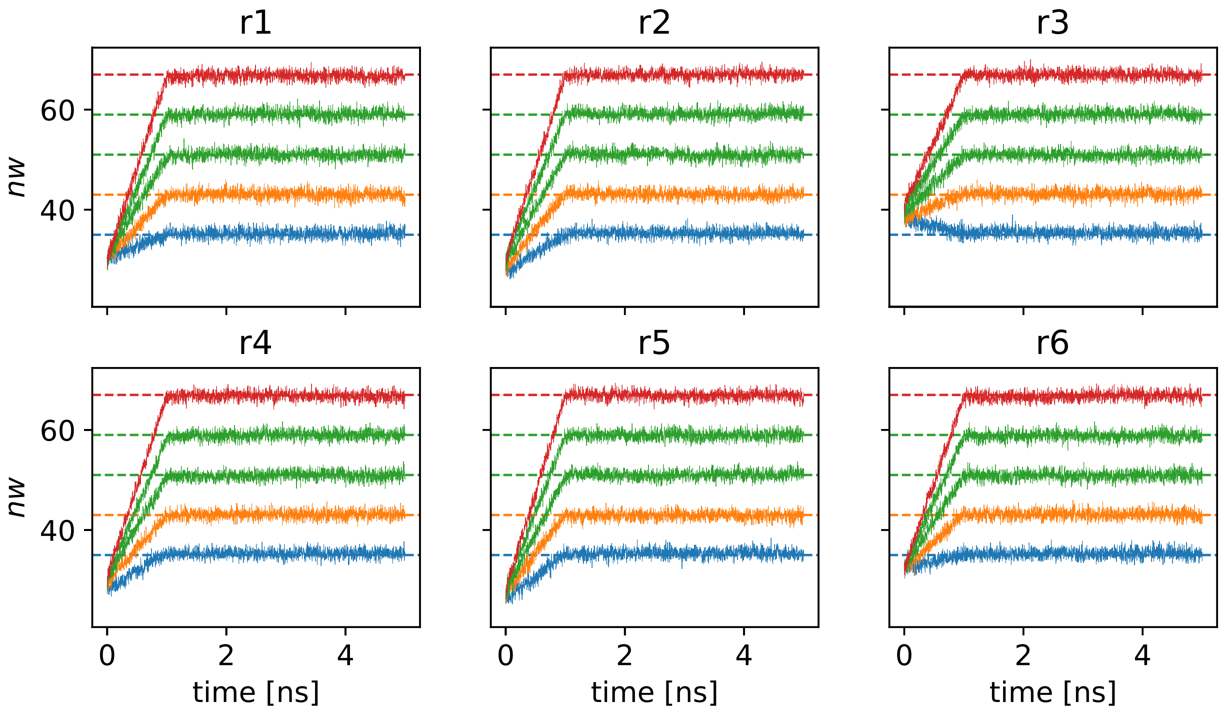

Fig. S7 Number of water molecules n_w sampled during some RMD simulations for the system shown in Fig. 4 of the main manuscript. Each trace represent the sampling under a given constraint target $n_{w,0}$, represented by dashed lines. The initial slope is the initial transient during which the target $n_{w,0}$ is updated from the initial unbiased value to the final one. Mean forces for each RMD are computed considering the sampling after 2 ns; standard error of the mean force is estimated by using a block average procedure, with the length of each block being 100 ps. Mean forces are integrated via simple euler method to obtain the free energy profile, and the error on the profile is computed by propagating the mean forces errors along the summation.

FIGURE S8 - RMD of the WT Ca^{2+}-free state (6V3G) without intruded lipids

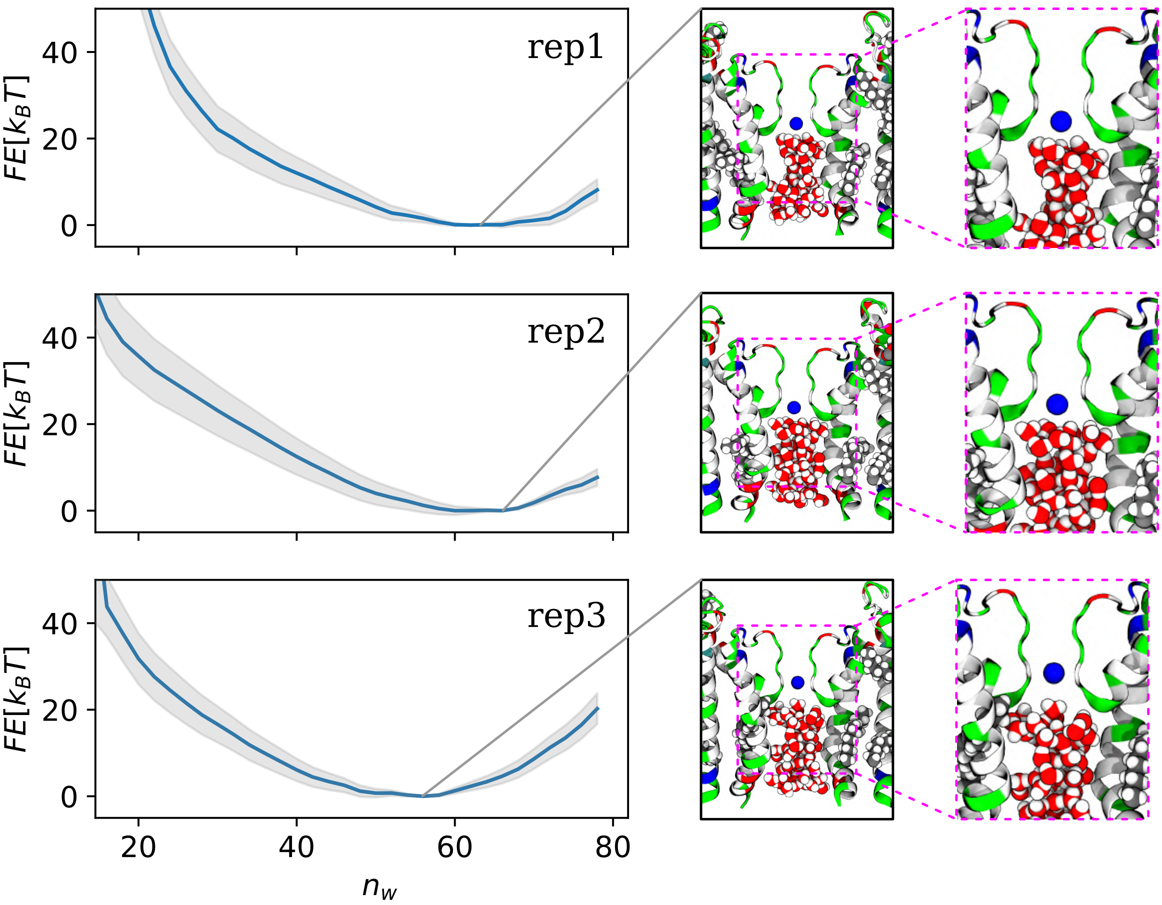

Fig. S8 Free energy profiles for three replica of the WT Ca^{2+}-free state before lipids intrude inside the DPV. The profiles show that for all the three replica it exist only one global minimum, corresponding to the wet state, while dry states (lower filling level, $n_w < 45$) are found to be not stable. This additional set of simulations largely support our hypothesis that lipid intrusion is the key feature to enable the pore gating. Lateral panels represents the last frame of the RMD trajectory corresponding to minimum of the free energy profile. Protein is displayed in New Cartoon representation, while water molecules and the potassium ion inside the selectivity filter are displayed in common VDW representation, rendered by using VMD [52].

FIGURE S9 - Comparison of TIP3P vs SPCE water model in RMD simulations

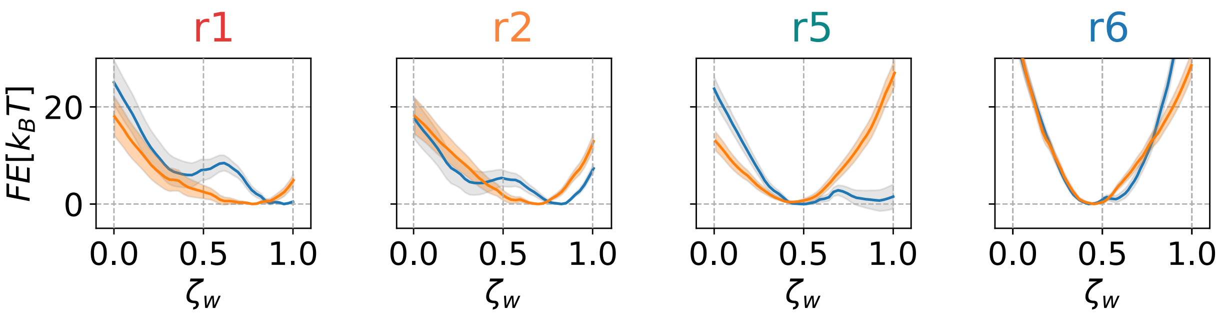

Fig. S9 Water filling free energy profiles computed via RMD simulations for the system reported in Fig. 4 of the main manuscript, computed also by using the TIP3P force field for the water, in combination with CHARMM36 force field for the protein and lipids, see orange lines. Blue lines are the same profile reported in the manuscript, representing the systems simulated with SPC/E water model in combination with Amber force field, see methods. Indeed, the most notable difference lie in the fact that orange lines do not show metastabilities in any of the simulated replicas. This fact is not particularly surprising, since TIP3P do not well reproduce the surface tension of the water. Another difference is that in the wet state TIP3P seems to display a lower density of water inside the control box. This is in line with previous reported works, where the lowering of density of water was correlated to the lower conductance of the closed state [24]. Neverthless, TIP3P predicted a wet minimum for replica $r1$ and $r2$, while a dry one for replicas $r5$ and $r6$, overall confirming the observation that the hydrophobic gating can easily happen in presence of lipid tail intruded into the DPV.

FIGURE S10 - RMD of the full-length Ca^{2+}-int without intruded lipids

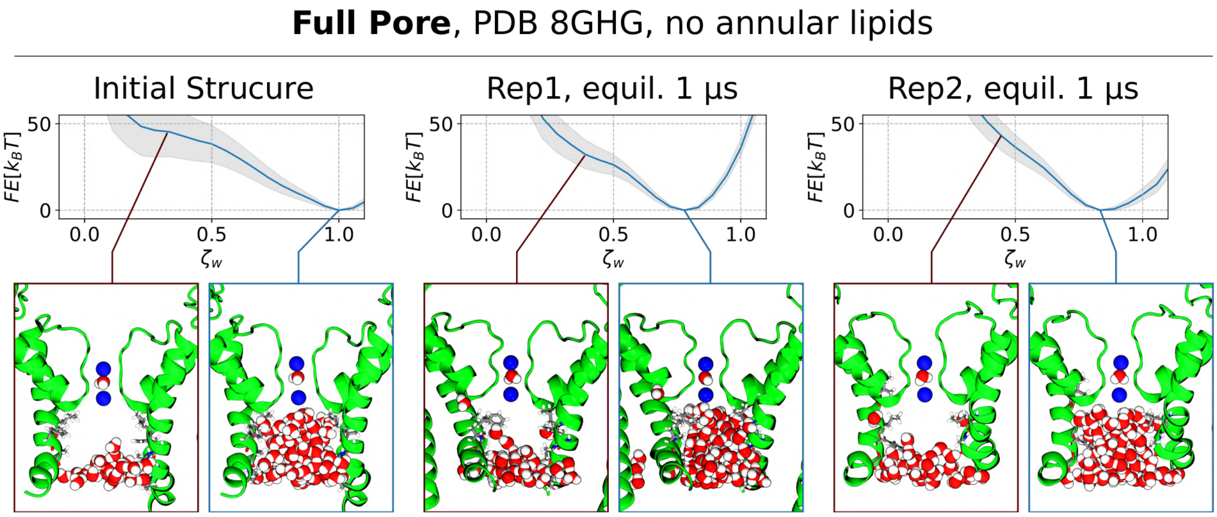

Fig. S10 Free energy profiles for three replica of the full 8GHG pore before lipids intrude inside the DPV. The profiles are computed using the initial structure and the last frame of the $1\mu s$ MD simulations reported in Supp. Fig. S2. The profiles show that for all the three replica it exist only one global minimum, corresponding to the wet state, while dry states (lower filling level, $n_w < 45$) are found to be not stable. This additional set of simulations largely support our hypothesis that lipid intrusion is the key feature to enable the pore gating. Sanpshots represents the last frame of the RMD trajectory corresponding to indicated states on the free energy profile. Protein is displayed in New Cartoon representation, while water molecules and the potassium ion inside the selectivity filter are displayed in common VDW representation, rendered by using VMD [52].

FIGURE S11 - Bottom view of Fig. 4c minima.

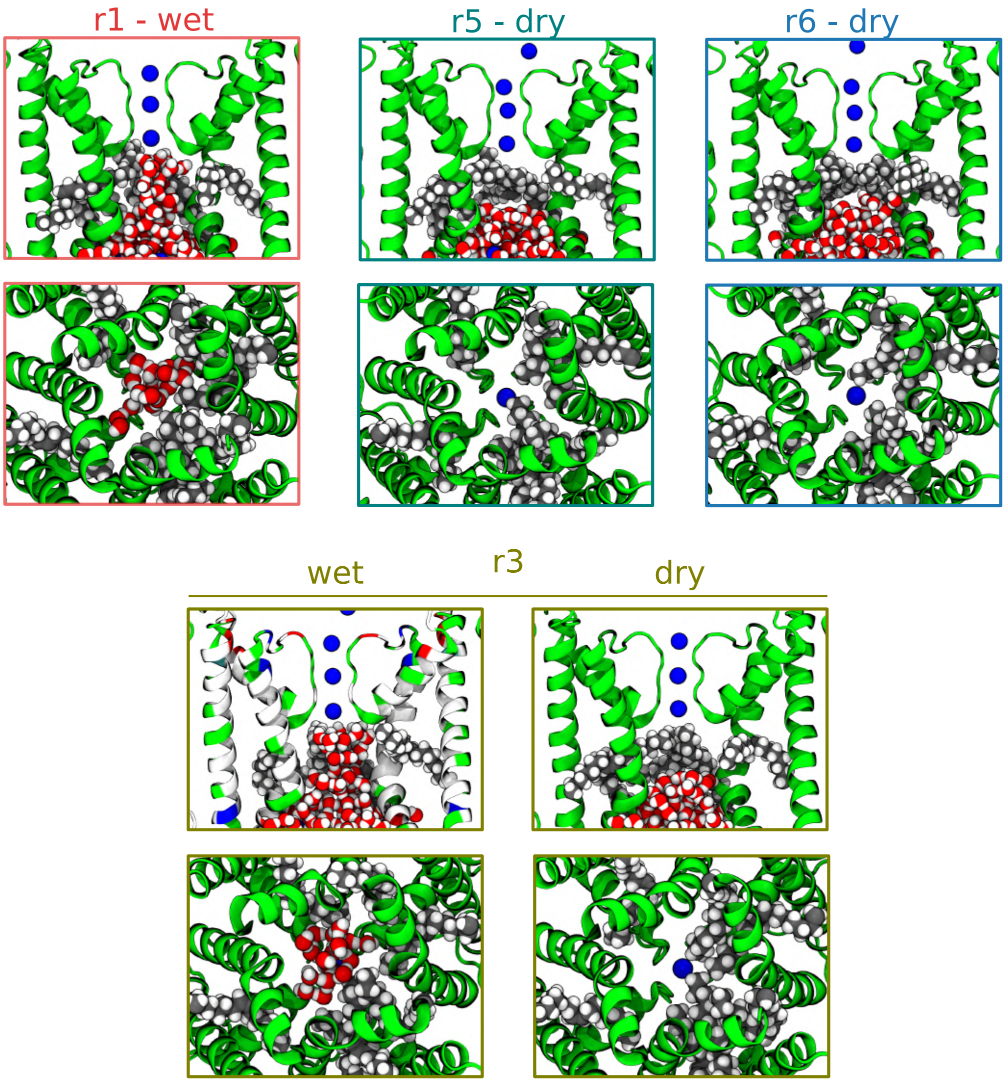

Fig. S11 Bottom view of the systems corresponding to panel c in Figure 4. The top row shows the systems r1 (wet), r5 (dry), and r6 (dry), while the bottom row focuses on the system r3 in both its wet and dry states. The protein is shown in green cartoon, with the intruded lipids in grey and white spheres. Water molecules, visible in the wet configurations, are represented in red and white spheres. Potassium ions also depicted in blue. These views complement the side perspectives shown in the main figure, providing additional structural details of the hydration and lipid organization through the pore inner cavity and the lateral fenestrations.

FIGURE S12 - Radii profiles of Fig. 4c minima, computed with HOLE.

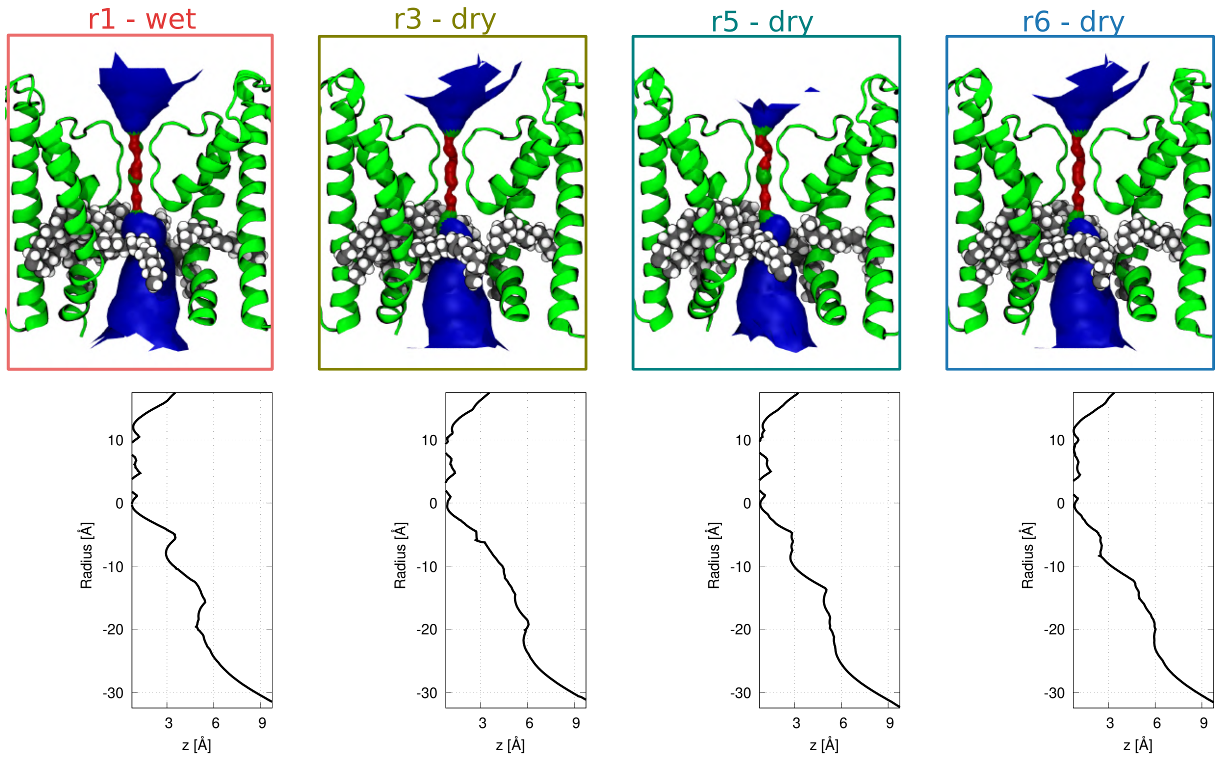

Fig. S12 Inner channel radius of the systems corresponding to panel c in Figure 4, computed with HOLE. The top row shows the 3D representation of the HOLE output computed on the systems r1 (wet), r3 (dry), r5 (dry), and r6 (dry). The bottom report the corresponding radii profile along z. These plots show how the lipid tails, among the selected states, progressively reduce the channel radius of the DPV, leading to a constriction of $\tilde{1}$nm length and radius lower than 3 Å just below the SF ($z = 0$Å to $z = -10$Å). This reduction of channel volume, together with the hydrophobicity of the lipid tails, enhances and stabilize the hydrohobic gating of the BK channel.
